# Editorial: Exploring the optimal endometrial preparation protocol for frozen-thawed embryo transfer

**DOI:** 10.3389/fendo.2024.1377488

**Published:** 2024-02-22

**Authors:** Ying Xu, Jing-Yan Song, Zhen-Gao Sun

**Affiliations:** ^1^The First Clinical College, Shandong University of Traditional Chinese Medicine, Jinan, China; ^2^Reproductive Center of Integrated Medicine, The Affiliated Hospital of Shandong University of Traditional Chinese Medicine, Jinan, China

**Keywords:** frozen embryo transfer, natural cycle, hormone replacement cycle, corpus luteum (CL), pregnancy outcomes

A successful frozen embryo transfer (FET) rate has gradually increased due to the continuous improvement of cryopreservation technology. The convenience of storing frozen embryos for an extended period allows patients to have more choice and flexibility. In recent years, FET has proven to be a more viable option than fresh embryo transfer (1). Studies have indicated that FET has a higher live birth rate (LBR) than fresh embryo transfer (2). While FET cycles are becoming increasingly popular, it remains unclear what is the best protocol for preparing the endometrium prior to this procedure. More and more studies have recently discussed the optimal endometrial preparation protocol.

## Obstetric and perinatal complications

The researchers who contributed to this Research Topic have published a total of 11 thematic articles, including 1 review, 2 meta-analyses, 7 original research articles, and 1 study protocol, which highlight optimal endometrial preparation protocols. Hormone replacement therapy (HRT) is currently the most widely used treatment due to its broad applicability and low cycle cancellation rate (3). Nevertheless, it is associated with an increased risk of complications during obstetrics and delivery (4). Three of the articles focused on obstetric and perinatal outcomes in different FET cycles, among which Epelboin et al. highlighted that prolonged doses of exogenous estrogen-progesterone may be harmful to gestational vascular pathologies, while the corpus luteum (CL) in ovulatory cycle (OC-FET) has a protective effect on the prevention of gestational vasculopathies. Also, Zhao et al. found that artificial cycles (AC-FET) have a negative impact on obstetric and perinatal complications, including gestational hypertension, preeclampsia, gestational diabetes mellitus, large for gestational age, macrosomia, placenta previa, small for gestational age, preterm labor, postpartum hemorrhage, placental abruption, and premature rupture of membranes at a premature pregnancy. Additionally, Wang et al. found that AC-FET may lead to abnormal placental attachment, which may result in obstetric complications such as preeclampsia, postpartum hemorrhage, and a higher incidence of cesarean delivery. A lack of CL may be responsible for this phenomenon in AC-FET, and the absence of CL could impair maternal hemodynamics and cardiovascular fitness for pregnancy in the first trimester, which leads to adverse pregnancy outcomes (e.g., preeclampsia) (5), as well as the fact that the CL produces a variety of vasoactive molecules, like relaxin, prorenin and other unknown molecules, which contribute to the global changes that occur during pregnancy and help reduce hypertensive disorders later (6, 7). Ultimately, precaution should be exercised whenever AC-FET is used, and specific approaches can be explored to mitigate the increased risk of obstetric and perinatal complications.

## Influencing factors of pregnancy outcomes

Several articles have explored which factors affect pregnancy outcomes. In a study conducted by Wang et al., it was found that atosiban can improve implantation rate, positive pregnancy rate, clinical pregnancy rate (CPR), and LBR in repeated implantation failure (RIF) patients, which may be attributed to atosiban’s beneficial effects on uterine contractility and endometrial receptivity (8–10). According to Liu et al., progesterone supplementation duration did not significantly affect AC-FET outcomes, and single-blastocyst transfers were recommended, as blastocysts have improved gestational outcomes than cleavage embryos because the blastocyst reselection process crosses developmental barriers and culls embryos with poor developmental potential (11, 12). In their study, Rodríguez-Varela et al. found that the duration of estrogen exposure prior to exogenous progesterone administration in HRT cycles did not affect clinical outcomes, allowing patients, physicians, and clinics to adjust dates optimally to meet their needs. As determined by Demirel et al., nearly half of NC-FETs require luteal phase support (LPS) because of low progesterone, and the authors also recommend that 25 mc subcutaneous progesterone salvages LPS and produces a similar ongoing pregnancy rate, and that LPS should be initiated prior to embryo transfer in all NC-FET cases. Data from Guler et al. report that higher serum luteinizing hormone (LH) levels are associated with higher CPRs and LBRs prior to initiation of FET with progestogens. Given that early initiation of estrogen therapy may be associated with lower LH levels prior to progestogen administration, delaying estrogen initiation to day 4 of the AC-FET cycle may be a favorable strategy to improve pregnancy outcomes. According to Huang et al., RNA-seq-based ER testing (rsERT) and preimplantation genetic testing (PGT) were both effective for improving ART efficacy, and endocrine dysregulation caused by multiple endocrine neoplasia type 1 (MEN1), rather than mutations in the MEN1 gene, was responsible for endometrial receptivity abnormalities. In this case, we demonstrate how treatment guidelines for patients suffering from MEN1 infertility can be developed by integrating multiple approaches, such as rsERT, PGT, and multidisciplinary team management.

## Final considerations

In summary, this Research Topic contributes to our understanding of optimal endometrial preparation regimens, and further studies are required to determine the optimal regimen and dosage of HRT supplementation for various estrogens and progestogens, as well as to identify specific strategies for preventing adverse outcomes in AC-FET cases that cannot be avoided.

## Author contributions

YX: Writing – original draft. J-YS: Conceptualization, Funding acquisition, Supervision, Writing – review & editing. Z-GS: Funding acquisition, Supervision, Writing – review & editing.
